# The Unique Antimicrobial Recognition and Signaling Pathways in Tardigrades with a Comparison Across Ecdysozoa

**DOI:** 10.1534/g3.119.400734

**Published:** 2020-01-22

**Authors:** Marc A. Mapalo, Kazuharu Arakawa, Caitlin M. Baker, Dennis K. Persson, Denise Mirano-Bascos, Gonzalo Giribet

**Affiliations:** * Museum of Comparative Zoology, Department of Organismic and Evolutionary Biology, Harvard University, 26 Oxford Street, Cambridge, MA 02138,; † Erasmus Mundus Master Programme in Evolutionary Biology (MEME),; ‡ Institute for Advanced Biosciences, Keio University, Tsuruoka, Yamagata 997-0052, Japan,; § Faculty of Environment and Information Studies, Keio University, Fujisawa, Kanagawa 252-0882, Japan, and; ** National Institute of Molecular Biology and Biotechnology, University of the Philippines, Diliman, Quezon City, Philippines 1101

**Keywords:** tardigrade immune response, ecdysozoan immune response, biotic stress response, Genetics of Immunity

## Abstract

Tardigrades are microscopic animals known to withstand unfavorable abiotic conditions. These animals are also constantly exposed to biotic stresses, including parasites and internal microbiomes. However, the tardigrade immune mechanisms against these biotic stresses are largely uncharacterized. Due to the contentious phylogenetic position of tardigrades, it is not intuitive whether they possess an immune system more similar to that of arthropods (*e.g.*, Toll, Imd, and JNK pathways of the *Drosophila melanogaster* antimicrobial response) or to that of nematodes (*e.g.*, the Tir-1/Nsy-1/Sek-1/Pmk-1/Atf-7 signaling cassette [called Tir-1 pathway here]) in *Caenorhabditis elegans*). In this study, comparative genomic analyses were conducted to mine homologs of canonical *D. melanogaster* and *C. elegans* immune pathway genes from eight tardigrades (*Echiniscoides* cf. *sigismundi*, *Echiniscus testudo*, *Hypsibius exemplaris*, *Mesobiotus philippinicus*, *Milnesium tardigradum*, *Paramacrobiotus richtersi*, *Richtersius* cf. *coronifer*, and *Ramazzottius varieornatus*) and four non-arthropod ecdysozoans (two onychophorans: *Epiperipatus* sp. and *Opisthopatus kwazululandi*; one nematomorph: *Paragordius varius*; and one priapulan: *Priapulus caudatus*) in order to provide insights into the tardigrade antimicrobial system. No homologs of the intracellular components of the Toll pathway were detected in any of the tardigrades examined. Likewise, no homologs of most of the Imd pathway genes were detected in any of the tardigrades or any of the other non-arthropod ecdysozoans. Both the JNK and Tir-1 pathways, on the other hand, were found to be conserved across ecdysozoans. Interestingly, tardigrades had no detectable homologs of *NF-κB*, the major activator of antimicrobial response gene expression. Instead, tardigrades appear to possess *NF-κB* distantly related *NFAT* homologs. Overall, our results show that tardigrades have a unique gene pathway repertoire that differs from that of other ecdysozoans. Our study also provides a framework for future studies on tardigrade immune responses.

Tardigrades are microscopic invertebrates known for their ability to withstand a range of extreme abiotic stresses, such as prolonged periods of desiccation and freezing, ionizing radiation, and the vacuum of space (*e.g.*, Jönsson *et al.* 2008; Guidetti *et al.* 2011). These animals are also susceptible to many biotic stresses, including infections from fungal parasites (Vecchi *et al.* 2016). These include *Ballocephala sphaerospora*, the first identified fungal parasite known to infect tardigrades. These fungi release spores which adhere to the tardigrade cuticle, bore through it, and release their contents internally (Drechsler 1951). The ciliate *Pyxidium tardigradum* is also known to specifically attach to limnoterrestrial eutardigrades, taking advantage of the host’s locomotion so it can constantly feed in new environments as the host moves around (Vicente *et al.* 2008). Tardigrades also harbor species-specific and environmentally dependent (*i.e.*, *in situ vs.* lab rearing) microbiomes (Vecchi *et al.* 2018). Several marine heterotardigrades host bacteria within specialized cephalic vesicles, potentially using substances these bacteria produce as secondary energy sources when food is limited (Vecchi *et al.* 2016). Given all these biological interactions, it is intuitive that tardigrades must have immune mechanisms to protect themselves from parasites or to manage their endosymbionts. Understanding the tardigrade immune system therefore provides another perspective on how this group of animals survives. However, there has been no published study on the immune response of tardigrades against these biotic stresses, to the best of our knowledge. Comparing tardigrades with closely related and genetically well-characterized animals, such as arthropods and nematodes, could provide insights into how tardigrades defend themselves against potential pathogens.

Tardigrades are members of the clade Ecdysozoa, composed of animals that undergo ecdysis or molting (Aguinaldo *et al.* 1997; see a recent review in Giribet and Edgecombe 2017). Within this clade are two of the best-studied animal model organisms – the insect *Drosophila melanogaster* and the nematode *Caenorhabditis elegans* – whose immune systems are well-characterized. Although they are both ecdysozoans, they differ substantially in their antibacterial and antifungal pathways.

In *D. melanogaster*, the two major regulators of the humoral immune response are the Toll and Imd pathways (Buchon *et al.* 2014). The Toll pathway is normally activated after infection by gram-positive bacteria and fungi while the Imd pathway is activated after infection by gram-negative bacteria. These pathways are activated when pathogen-associated molecular patterns (PAMPs) are recognized by pattern recognition molecules, such as peptidoglycan recognition proteins (PGRPs). These ultimately lead to the activation of NF-κB transcription factors (Dorsal and Dif in the Toll pathway and Relish in the Imd pathway) which are involved in further activation of immune effector molecules, such as antimicrobial proteins (Valanne *et al.* 2011; Myllymäki *et al.* 2014). These two pathways are also known to synergistically work together in controlling the activation of these antimicrobial proteins (Tanji *et al.* 2007). In the Imd pathway, the Tak1 protein can also activate the JNK pathway which stimulates a stress response and antimicrobial protein expression. This pathway is composed of tyrosine kinases and activates a heterodimeric transcription factor composed of Jun-related transcription factor (Jra) and Kayak (Fos) (Delaney *et al.* 2006; Valanne *et al.* 2011).

On the other hand, NF-κB homologs are absent in *C. elegans* and their Toll pathway gene homologs do not have direct anti-bacterial functional roles. Instead, the Tir-1/Nsy-1/Sek-1/Pmk-1/Atf-7 signaling cassette (called the Tir-1 pathway in this paper), which is also composed of tyrosine kinases, is involved in the *C. elegans* immune response (Ermolaeva and Schumacher 2014).

Tardigrades are often grouped with arthropods and onychophorans in the clade Panarthropoda (*e.g.*, Nielsen 2011; Giribet and Edgecombe 2017). However, phylogenetic analyses of large datasets often cluster tardigrades with nematodes (*e.g.*, Borner *et al.* 2014; Yoshida *et al.* 2017) or are inconsistent depending on analytical conditions (Laumer *et al.* 2019). Given this uncertain phylogenetic position, it is not easy to predict whether tardigrades would have a gene repertoire for immune response more similar to that of *D. melanogaster* or *C. elegans*.

In this study, we used genomic and transcriptomic sequence data from both of these model organisms and identified homologs of *D. melanogaster* and *C. elegans* antimicrobial immune pathway genes in assembled tardigrade genomes and transcriptomes to reconstruct parts of the antimicrobial gene repertoire of the tardigrade immune system (*i.e.*, in which ways it is similar to or different from those of the two best-characterized animal model organisms). For this, we used eight tardigrade species that represent four orders and seven families based on the new taxonomy proposed by Guil *et al.* (2019): two heterotardigrades: *Echiniscoides* cf. *sigismundi* (Echiniscoidea: Echiniscoididae) and *Echiniscus testudo* (Echiniscoidea: Echiniscidae), one apotardigrade: *Milnesium tardigradum* (Apochela: Milnesiidae), and five eutardigrade: *Hypsibius exemplaris* (Hypsibioidea: Hypsibiidae; formerly referred to as *Hypsibius dujardini*; Gasiorek *et al.* 2018), *Ramazzottius varieornatus* (Hypsibioidea: Ramazzottiidae), *Mesobiotus philippinicus* (Macrobiotoidea: Macrobiotidae), *Paramacrobiotus richtersi* (Macrobiotoidea: Macrobiotidae), and *Richtersius* cf. *coronifer* (Macrobiotoidea: Richtersiidae). To further understand the diversity of immune responses within Ecdysozoa, we also identified homologs in assembled genomes and transcriptomes of species from three other ecdysozoan phyla. In order to provide a more comprehensive view of Panarthropoda, we included two onychophorans, one from each of the two extant families: *Epiperipatus* sp. (Peripatidae) and *Opithopatus kwazululandi* (Peripatopsidae). We also included the nematomorph *Paragordius varius* to represent the other phylum in the clade Nematoida. Lastly, we included the priapulan *Priapulus caudatus* as a representative of Scalidophora and used it as a control to check the stringency of our homolog detection method. To identify homologs, we used a step-by-step approach of combining sequence similarity searches, detection of conserved domains, and phylogenetic reconstruction. This provided us with a list of putative homologs that not only share sequence similarity with *D. melanogaster* and *C. elegans* immune pathway genes but also contain domains that are considered functionally important in the immune response. This study constitutes the first comprehensive search of antimicrobial immune genes in Tardigrada, Nematomorpha, and Priapulida, thus enabling us to understand the diversity of the ecdysozoan immune responses.

## Materials and Methods

### Sample Dataset

For the tardigrade dataset, we used two novel transcriptome assemblies (*Mesobiotus philippinicus* and *Echiniscus testudo*). The transcriptome of *M. philippinicus* was obtained from a single tardigrade sample using a modified method described in Arakawa *et al.* (2016), while the transcriptome of *E. testudo* was assembled using 20 individually-sequenced tardigrade samples (see File S1 for detailed methods of sample collection and library preparation of the tardigrade samples). We also obtained two transcriptome raw read files (Kamilari *et al.* 2019) from the NCBI SRA database (https://www.ncbi.nlm.nih.gov/sra/): *Echiniscoides* cf. *sigismundi* (SRX421163), *Richtersius* cf. *coronifer* (SRX4213802); two transcriptome assemblies from NCBI TSA database (https://www.ncbi.nlm.nih.gov/genbank/tsa/): *Milnesium tardigradum* (GFGZ00000000.1), *Paramacrobiotus richtersi* (GFGY00000000.1); and sequences from two proteomes (Yoshida *et al.* 2017) from the Tardigrade Ensembl (http://ensembl.tardigrades.org/index.html): *Hypsibius exemplaris* (*Hypsibius dujardini* nHD.3.1.5.proteins), and *Ramazzottius varieornatus* (Rv101.proteins). For the onychophoran dataset, we used two novel onychophoran transcriptome assemblies of *Epiperipatus* sp. and *Opisthopatus kwazululandi* (see File S1 for detailed methods of sample collection and library preparation of the onychophoran samples). Lastly, raw transcriptome reads of one nematomorph (*Paragordius varius* NCBI SRA: ERX1879698) and the proteome sequences of one priapulan (*Priapulus caudatus* NCBI Genome: Priapulus_caudatus-5.0.1) were obtained from online databases.

The quality of the raw tardigrade and nematomorph transcriptome reads were first checked using Fastqc v0.11.5 and random sequencing errors were corrected using the k-mer based method Rcorrector v1.0.2 (Song and Florea 2015). After removing “unfixable reads” identified by Rcorrector, the remaining reads were trimmed using default settings in TrimGalore! v0.3.7 or v0.5.0 (https://www.bioinformatics.babraham.ac.uk/projects/trim_galore/). Trimmed reads were aligned against small and large ribosomal RNAs (rRNAs), and tardigrade mitochondrial sequences (74 single-genes and two complete mitochondrial genomes, see File S2) obtained from the SILVA non-redundant database release 128 (Quast *et al.* 2013) and NCBI database, respectively, using Bowtie2 v2.2.2 (Langmead and Salzberg 2012). Finally, the unaligned reads were used for a *de novo* transcriptome assembly using a no_normalize_reads parameter in the Trinity assembler v2.3.2. Assembly statistics were also obtained using the Trinity stats option (Table S1). For the onychophoran transcriptome reads, the same steps were used, but were aligned against rRNA and Panarthropoda mitochondrial sequences obtained from the SILVA and MetAMIGA databases, respectively. The same alignment step was also done after assembling the onychophoran transcriptomes.

A BUSCO v3.0.2 (Simão *et al.* 2015) analysis against the Metazoa dataset was conducted for all transcriptomes to assess the completeness of the assemblies (Table 1). For the proteome sequences, their corresponding transcriptome sequences were obtained from the same databases and used for the BUSCO analysis. Candidate protein coding sequences for each transcriptome were predicted using the default settings of TransDecoder v3.0.0 or v5.3.0. CD-Hit v4.6.4 (Li and Godzik 2006) was used for each protein dataset with a 95% clustering threshold to decrease redundancy of the protein sequences.

**Table 1:** Results of BUSCO analyses using the Metazoa dataset (978 Total BUSCOs)

		Complete BUSCOs (Single;Duplicated)	Fragmented BUSCOs	Missing BUSCOs
***Echiniscoides* cf. *sigismundi***	number of genes	801 (104;697)	37	140
	*percentage*	*81.90 (10.63;71.27)*	*3.78*	*14.31*
***Echiniscus testudo***	number of genes	739 (313;426)	130	109
	*percentage*	*75.56 (32.00;43.56)*	*13.29*	*11.15*
***Hypsibius exemplaris***	number of genes	874 (841;33)	22	82
	*percentage*	*89.37 (85.99;3.37)*	*3.37*	*2.25*
***Mesobiotus philippinicus***	number of genes	708 (48;660)	51	219
	*percentage*	*72.39 (4.91;67.48)*	*5.21*	*22.39*
***Milnesium tardigradum***	number of genes	594 (419;175)	176	208
	*percentage*	*60.74 (42.84;17.89)*	*18.00*	*21.27*
***Paramacrobiotus richtersi***	number of genes	862 (221;641)	30	86
	*percentage*	*88.14 (22.60;65.54)*	*3.07*	*8.79*
***Ramazzottius varieornatus***	number of genes	867 (836;31)	26	85
	*percentage*	*88.65 (85.48;3.17)*	*2.66*	*8.69*
***Richtersius* cf. *coronifer***	number of genes	863 (26;837)	34	81
	*percentage*	*88.24 (2.66;85.58)*	*3.48*	*8.28*
***Epiperipatus* sp.**	number of genes	759 (553;206)	194	25
	*percentage*	*77.61 (56.54;21.06)*	*19.84*	*2.56*
***Opisthopatus kwazululandi***	number of genes	808 (356;452)	149	21
	*percentage*	*82.62 (36.40;46.22)*	*15.24*	*2.15*
***Paragordius varius***	number of genes	834 (105;729)	34	110
	*percentage*	*85.28 (10.74;74.54)*	*3.48*	*11.25*
***Priapulus caudatus***	number of genes	907 (676;231)	29	42
	*percentage*	*92.74 (69.12;23.62)*	*2.97*	*4.29*

### Gene Mining of Immune-Related Genes

Protein sequences of arthropod orthologs of *D. melanogaster* and nematode orthologs of the *C. elegans* immune genes (Table S2) were first obtained from OrthoDB v9 (Zdobnov *et al.* 2017) and ImmunoDB (Waterhouse *et al.* 2007). Each ortholog gene dataset was aligned using MAFFT v7.313 (Katoh and Standley 2013) and used to build profile hidden Markov models (HMMs) using HMMER v3.1 (Mistry *et al.* 2013). The profile HMMs were used as queries to obtain the first set of candidate homologs of specific immune genes using the Easel application of HMMER. Then, a reciprocal hit search using BLAST v2.2.30 (Camacho *et al.* 2009) was done by using the longest protein isoform sequence of a *D. melanogaster* or *C. elegans* immune gene obtained from Flybase and Wormbase, respectively, as query (Table S3) in a BLASTp search against the first set of candidate genes as database. Hits with an e-value less than 10^−6^ were retained and formed the second set of candidate genes. This set was then used as query in an online BLASTp search (https://blast.ncbi.nlm.nih.gov/Blast.cgi) against the *D. melanogaster* or *C. elegans* protein database. The third set of candidate genes were composed of genes that showed the specific *D. melanogaster* or *C. elegans* immune gene as the top hit and had a bit score greater than 80, a percent identity greater than 20%, and e-value less than 10^−6^ (based on Palmer and Jiggins 2015). Finally, the protein domains of these genes were determined using the NCBI Batch Conserved Domain tool (https://www.ncbi.nlm.nih.gov/Structure/bwrpsb/bwrpsb.cgi) to check whether they contained the domains of the *D. melanogaster* or *C. elegans* immune genes with the corresponding domain identifiers (Table S4). This was also done to quantify the gene homolog numbers if there were multiple candidate genes from the third set. Different genes were considered as different paralogs if they shared the same protein domains (*e.g.*, both genes encode for an LRR domain), and they were counted as one gene if their protein domains were parts of a whole gene (*e.g.*, one gene encodes for an LRR domain while the other encodes for a TIR domain; see File S3).

Since the Trinity assembler can predict and provide different putative isoform sequences of a single gene, a gene can be represented by multiple sequences. Thus, the longest isoform was used as the representative sequence of a given gene. *Caenorhabditis elegans* genes were identified as homologs of *D. melanogaster* immune genes if they were the corresponding top hits in the homology section of Flybase and Wormbase. The same method was used for identifying *D. melanogaster* homologs of *C. elegans* immune genes.

### Structural and Phylogenetic Analyses

LRR domains of the Toll homologs were determined using the LRRfinder (http://www.lrrfinder.com/, Offord *et al.* 2010). We used TMHMM (http://www.cbs.dtu.dk/services/TMHMM/, Krogh *et al.* 2001) to detect the presence of transmembrane domains in Toll homologs. Protein sequences of the TIR domain were obtained using SMART domain search (http://smart.embl-heidelberg.de/smart/set_mode.cgi?NORMAL=1, Letunic and Bork 2018).

For the Toll phylogenetic analysis, we used protein sequences of the TIR domains of the putative TIR-containing Toll homologs obtained from our gene mining search, together with TIR domain sequences of Toll homologs in Palmer & Jiggins (2015) and nematode Toll homologs from WormBase. For the NF-κB/NFAT phylogenetic analysis, we used protein sequences from the first sets of candidate genes that contain the Rel-homology domain (RHD) (see File S4), together with NF-κB and Nuclear factor of activated T-cells (NFAT) homologs of animals from the following phyla: Porifera, Cnidaria, Arthropoda, Mollusca, Annelida, Echinodermata, and Chordata (Table S5) obtained from ImmunoDB and OrthoDB v9.

All sequences were aligned using MAFFT v7.313 (see Files S5 and S6). Alignments were not trimmed since some of the sequences were incomplete. The phylogenetic trees were built using the best-fit model obtained under the Akaike Information Criterion in ProtTest v3.4 (Darriba *et al.* 2011) with 1000 bootstrap replicates using the RAxML-HPC2 on XSEDE tool (Stamatakis 2014) available in CIPRES Science Gateway site (https://www.phylo.org/; Miller *et al.* 2010). All phylogenetic trees were viewed and edited in FigTree v1.4.3 (Rambaut 2016).

### Data Availability

The raw transcriptome reads were deposited in NCBI SRA: *M. philippinicus* (PRJNA509138), *E. testudo* (SAMN10601501-SAMN10601521), *Epiperipatus* sp. (SRP173483), and *Opisthopatus kwazululandi* (SRP173472). All Trinity transcriptome assemblies were deposited in Harvard Dataverse (https://doi.org/10.7910/DVN/CFNUGF). Supplemental material available at figshare: https://doi.org/10.25387/g3.9864353.

## Results and Discussion

### Tardigrades have Drosophila-Like Toll-Like receptors

**Figure 1:**
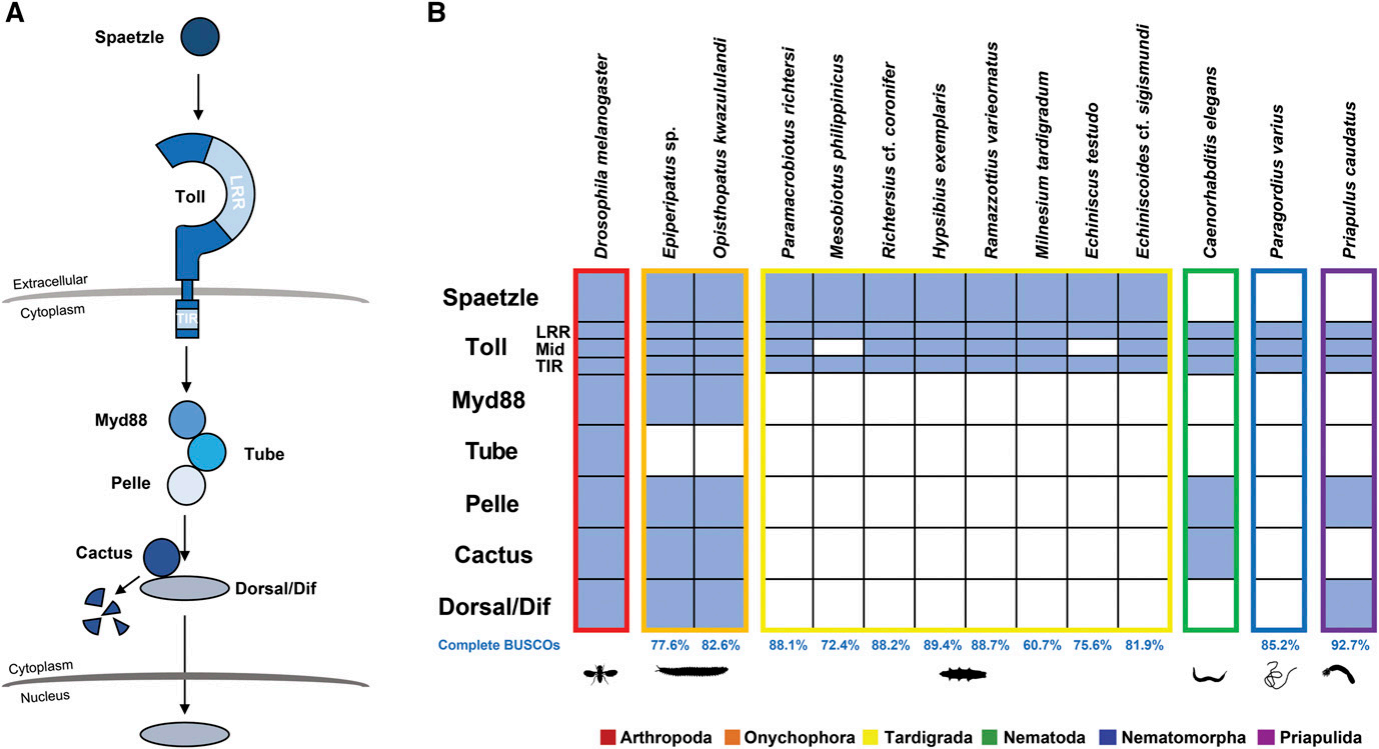
Tardigrade and other ecdysozoan Toll pathway gene homologs. (A) The *Drosophila melanogaster* Toll pathway. (B) The presence (filled boxes) and absence (empty boxes) of *D. melanogaster* Toll pathway gene homologs in tardigrades and other ecdysozoans. LRR – extracellular leucine rich repeat domain; Mid – transmembrane domain; TIR – intracellular domain.

One of the key components of the *D. melanogaster* Toll pathway (Figure 1a) is the transmembrane receptor Toll. During gram-positive bacterial and fungal infections, it is bound by a cleaved version of Spaetzle, which then leads to the activation of the whole pathway (Lemaitre *et al.* 1996; Valanne *et al.* 2011). Toll-like receptors (TLRs) have three distinct domains: the leucine-rich repeat (LRR) motifs in the extracellular region, the transmembrane domain, and the Toll/interleukin-1 receptor (TIR) (Bell *et al.* 2003). Members of this family are divided into two major structural types depending on the number of cysteine clusters in the LRR region. Most deuterostomes (including humans) have single cysteine cluster TLRs (sccTLRs), at the C-terminal end (CF motif; LRRCF), right next to the plasma membrane. Most protostomes (including *D. melanogaster* and *C. elegans*), on the other hand, have multiple cysteine cluster TLRs (mccTLRs), which possess two or more CF motifs and another cysteine cluster at the N-terminal end of LRR (NF motif; LRRNF) (Leulier and Lemaitre 2008). Cnidarians, an outgroup to bilaterians, are currently the most distantly related metazoans known to possess TLR homologs complete with the three domains and most of these homologs are mccTLRs. Because of this, it is hypothesized that the ancestral TLR emerged before the split of the bilaterians from cnidarians (Voogdt and van Putten 2016) and was of the mccTLR type (Brennan and Gilmore 2018).

**Figure 2:**
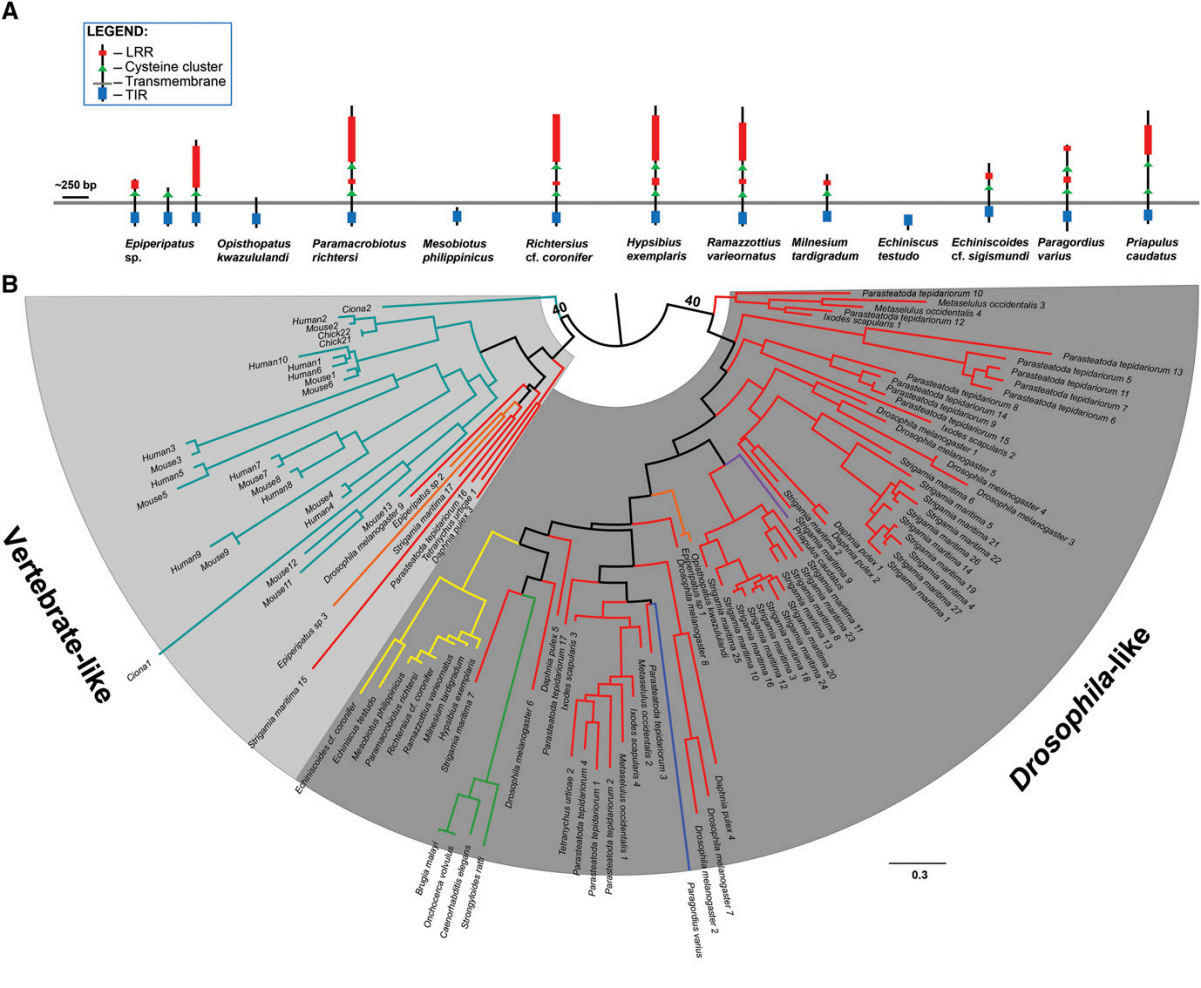
Tardigrade and other ecdysozoan Toll-like receptor homologs. (A) Predicted structure of TIR-containing *D. melanogaster* Toll receptor homologs of tardigrades and other ecdysozoans. (B) Phylogenetic tree of TIR domains of the Toll receptor homologs of tardigrades, other ecdysozoans, and vertebrates built using the LG+G model with 1000 bootstrap replicates and midpoint-rooted. Complete bootstrap values available in Figure S1. Branch colors represent different phyla. Scale bar = number of nucleotide substitution/site.

Our homology search confirmed the presence of transcripts encoding putative homologs of TLRs in tardigrades. However, protein domain analyses of the translated transcripts showed that only *E*. cf. *sigismundi*, *H. exemplaris*, *M. tardigradum*, *P. richtesi*, *R*. cf. *coronifer*, and *R. varieornatus* have homologs containing all three domains. *E. testudo* and *M. philippinicus* have homologs for the LRR and TIR domains (Table S6) but appear to have no transcripts coding for the transmembrane domain in the dataset (Figure 1b). Protein domain analyses also showed that all complete tardigrade TLRs are mccTLRs, similar to those of *D. melanogaster*, except for *E*. cf. *sigismundi* and *M. tardigradum* which showed sccTLR configurations (Figure 2a). Phylogenetic analysis using the TIR domains of the tardigrade TLRs, however, still clustered these two TLRs with the other tardigrade mccTLRs. This same phylogenetic pattern was also observed for the predicted TIR-only proteins of *E. testudo* and *M. philippinicus* (Figures 2b and S1 with bootstrap values). Thus, for those species that showed missing elements of the TLR, these sequences could have just been fragmented and were not represented completely in the dataset that we used. Indeed, BUSCO analysis showed that *E. testudo*, *M. philippinicus*, and *M. tardigradum* had the highest percentage of fragmented BUSCOs among all the tardigrade samples (Table 1). The *E*. cf. *sigismundi* transcriptome, however, had fewer fragmented BUSCOs (Table 1) suggesting that the sccTLR configuration observed in this species may not be artifactual. In addition, it should be noted that LRR-only and TIR-only containing proteins in Cnidaria (Bosch *et al.* 2009) and Porifera (Wiens *et al.* 2007) are still observed to be involved in immunity suggesting a conserved role for these proteins in the immune systems of Metazoa.

Putative TLR homologs were also found in the transcriptomes of the other three ecdysozoan groups. Protein domain analyses showed that TLR homologs of *P. varius* and *P. caudatus* are mccTLRs while *Epiperipatus* sp. are sccTLRs. However, *O. kwazululandi* does not have TLR homologs with the three domains. It had either LRR-only homologs or a TIR plus transmembrane-containing homolog. (Figure 2a, Table S6). It is also worth noting that the onychophoran *Epiperipatus* sp. has three complete TLR homologs (Table S6). Phylogenetic analysis clustered the nematomorph, priapulan, and *O. kwazululandi* TLR homologs with other mccTLRs (Figure 2b). For *Epiperipatus* sp., two homologs clustered with vertebrate sccTLRs while the last one clustered with mccTLRs despite having an sccTLR form (Figure 2a). Thus, as noted for tardigrades, these sequences could also have been fragmented in the transcriptome and may therefore be incomplete. Indeed, the onychophoran transcriptomes showed high percentage fragmented BUSCOs compared to other samples (Table 1).

### Spaetzle homologs were only detected in panarthropods

Mammalian TLRs are activated by the direct binding of pathogens to the LRR domain (Hopkins and Sriskandan 2005). The activation of the canonical *D. melanogaster* Toll pathway, on the other hand, is indirect since the Toll protein is activated by the binding of the endogenous ligand Spaetzle which is cleaved upon pathogen infection (Weber *et al.* 2003). In our homology search, we were only able to detect putative homologs of *Spaetzle* in the tardigrade and onychophoran samples. The apparent absence of *Spaetzle* homologs in our dataset, however, could be caused by the high stringency of our homology search. Alternatively, the TLRs of the other ecdysozoans could either bind to pathogen-associated molecules directly or may be activated by other ligands. Direct engagement of the TLRs to PAMPs has actually been observed in other invertebrates, such as cnidarians (Brennan *et al.* 2017) and mollusks (Wang *et al.* 2015). Direct activation of the TLR pathway by pathogen binding has also been observed in the Kuruma shrimp, *Marsupenaeus japonicus* (Sun *et al.* 2017). Therefore, we hypothesize that TLRs generally have the capability to directly engage pathogens, regardless of whether they are mccTLRs or sccTLRs. We further hypothesize that activation of the TLR pathway via the endogenous ligand Spaetzle could have originated only within the panarthropods, or potentially just within hexapods, even though Spaetzle proteins are also present in non-hexapod panarthropods.

To further explore this hypothesis, we looked at the presence of upstream genes in the *D. melanogaster* Toll pathway pathogen recognition that trigger Spaetzle cleavage. Upon fungal and gram-positive bacterial infection, extracellular proteins called Gram-negative binding proteins (GNBPs) and peptidoglycan recognition proteins (PGRPs) recognize and bind to these pathogens. GNBP3 is involved in fungal recognition (Gottar *et al.* 2006) while the GNBP1-PGRP-SA complex (Wang *et al.* 2006) and PGRP-SD (Bischoff *et al.* 2004) are involved in gram-positive bacterial recognition. In our homolog search, we were not able to detect putative *PGRP-SA/SD* and *GNBP1/3* homologs in any samples (data not shown). This suggests that the Spaetzle-mediated Toll pathway activation during pathogen infection could have originated only within hexapods since the molecules involved in Spaetzle cleavage were present only within this group.

### Tardigrades lack gene homologs of the canonical D. melanogaster antimicrobial NF-κB pathways

NF-κB proteins are a superfamily of transcription factors which contain a highly conserved Rel-homology domain (RHD) in their N-terminal sequences required for DNA binding, dimerization, and nuclear localization. Pathways that lead to the nuclear translocation of these proteins are called NF-κB pathways (Gilmore and Wolenski 2012). In *D. melanogaster*, two types of NF-κB pathways, the Toll and Imd pathways, are involved in the regulation of antimicrobial responses (De Gregorio *et al.* 2002). Upon fungal and Gram-positive bacterial infection, the Toll pathway activates the NF-κB transcription factors Dif and Dorsal. The Imd pathway, on the other hand, activates the NF-κB transcription factor Relish upon Gram-negative bacterial infection. All these transcription factors then activate the expression of antimicrobial peptide (AMP) effector genes, sometimes working synergistically (Tanji *et al.* 2007; Gilmore and Wolenski 2012).

Palmer and Jiggins (2015) showed that most of the genes involved in the *D. melanogaster* Toll pathway are present across Arthropoda. Gene mining results (Figure 1b) showed that this gene repertoire conservation is extended to both onychophorans *Epiperipatus* sp and *O. kwazululandi*. Tardigrades, however, show a different pattern compared to the other panarthropods. Although homologs of *Spaetzle* and *Toll* were present in tardigrades, no homologs of the intracellular components of the Toll pathway were detected, including the transcription factors *Dorsal* and *Dif*. Interestingly, the same apparent lack of the intracellular gene repertoire was also observed in the nematomorph *P. varius*. The priapulan *P. caudatus*, on the other hand, possesses a *Dorsal* homolog, as well as a homolog of the intracellular component *Pelle*.

The pattern observed in the tardigrades wherein homologs of most of the Toll pathway genes were not detected may be explained by at least one of the following reasons: First, all these genes may be truly absent in the tardigrades included in the analysis. This would indicate that the tardigrade immune defense does not involve the canonical *D. melanogaster* Toll pathway. However, the absence of these genes can only be proven after the genomes of these tardigrades are completely sequenced. It is worth noting, however, that the *H. exemplaris* and *R. varieornatus* proteomes used in this study came from the only two published tardigrade genomes and represent the most complete transcriptomes within the phylum. Second, the homologs of the intracellular components of the Toll pathway could have a high substitution rate so that their sequences would be very divergent from their arthropod homologs. However, Song *et al.* (2012) showed in their network-level molecular evolutionary analyses that the downstream genes in the animal Toll pathway evolve slowly and are more conserved than the upstream genes. If homologs of the downstream genes were present in the tardigrades, at least one of them should have been detected. Third, the gene mining pipeline could have been too stringent for detecting the tardigrade homologs. However, a *Dorsal* homolog was detected in *P. caudatus*, which is more distantly related to *D. melanogaster* than tardigrades. Thus, in theory, *Dorsal* homologs should have been detected if present in tardigrades—unless they exhibited unusually fast evolutionary rates. Lastly, the missing homologs may only be expressed at significant levels during a microbial infection and may therefore be missing from the current transcriptomic data, since all samples were believed to be uninfected at the time of sequencing. However, as stated earlier, the Toll pathway is the main regulator of the immune response and thus components of this pathway should be constitutively expressed so that they can activate immune effector genes quickly in case of infection. Taken together, the most likely explanation for the apparent lack of the components of the canonical Toll pathway at present is that tardigrades may have a different Toll pathway signaling axis when compared to other panarthropods.

**Figure 3:**
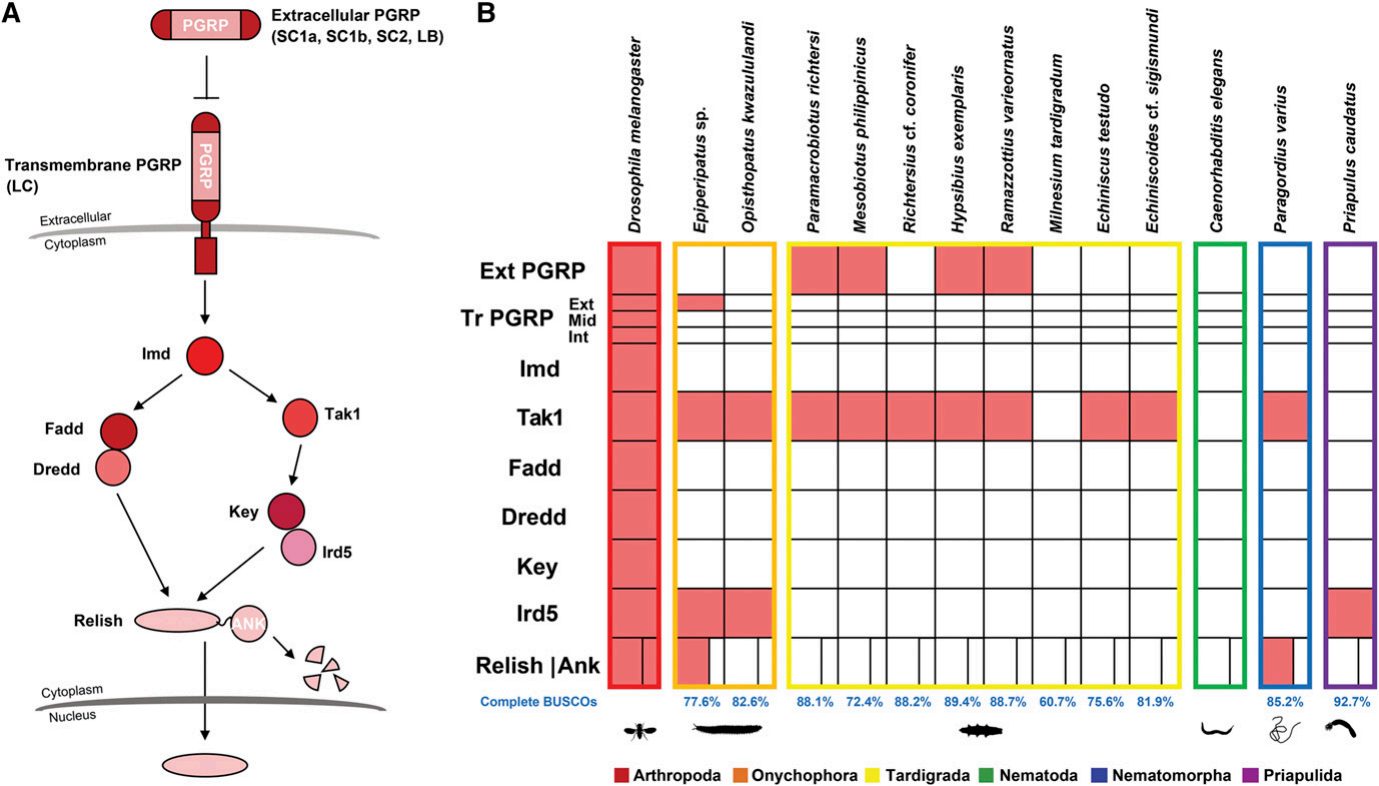
Tardigrade and other ecdysozoan Imd pathway gene homologs. (A) The *Drosophila melanogaster* Imd pathway. (B) The presence (filled boxes) and absence (empty boxes) of *D. melanogaster* Imd pathway gene homologs in tardigrades and other ecdysozoans. Ext PGRP – extracellular PGRP; Tr PGRP – transmembrane PGRP; Ext – extracellular domain; Mid – transmembrane domain; Int – intracellular domain; Ank – ankyrin repeats.

The other NF-κB pathway, the Imd pathway (Figure 3a), was shown to be more conserved within Mandibulata (=Myriapoda + Pancrustacea). This is due to the absence of homologs of some of the Imd pathway genes in Chelicerata, especially the Imd protein (Palmer and Jiggins 2015; Lai and Aboobaker 2017). Gene mining results showed that *Epiperipatus* sp. has a similar pattern as chelicerates, as it also has a *Relish* homolog but lacks an *Imd* homolog. *Opisthopatus kwazululandi*, meanwhile, lacks *PGRP* and *Relish* homologs (Figure 3b). Tardigrades yet again showed a different repertoire of Imd pathway genes when compared to the other panarthropods. Only *Tak1* homologs were detected in all tardigrades, except for *M. tardigradum*, while *PGRP* homologs were only detected in eutardigrades except for *R*. cf. *coronifer*. Furthermore, no homologs of the transcription factor *Relish* were detected in any tardigrade. For the other ecdysozoans, *P. varius* shares the same gene repertoire as the heterotardigrades and *R*. cf. *coronifer*, except that it has a *Relish* homolog. *Priapulus caudatus*, on the other hand, has a *Relish* homolog and a homolog of the intracellular component *Ird5*.

The absence of Toll pathway gene homologs in tardigrades and other ecdysozoans indicates that the canonical *D. melanogaster* Toll pathway might have originated and possibly became functional only before the split of arthropods and onychophorans. The absence of some of the Imd pathway genes in the chelicerates and the rest of the ecdysozoans indicates that the canonical *D. melanogaster* Imd pathway might have originated and possibly become functional only after the split of Mandibulata and Chelicerata. This could explain why the canonical *D. melanogaster* pathway was never found to be involved in the *C. elegans* antibacterial pathway (Irazoqui *et al.* 2010).

### Tardigrades do not have NF-κB homologs

The RHD-containing NF-κB superfamily is divided into two subfamilies. The first, called the NF-κB proteins, includes Relish in *D. melanogaster* and p100 and p105 in vertebrates. Most members of this subfamily are characterized by a C-terminal inhibitory ankyrin repeat that needs to be cleaved to activate the transcription factor. The other subfamily is composed of the Rel-like proteins and includes Dorsal and Dif in *D. melanogaster* and c-Rel, RelA, and RelB in vertebrates. Unlike the former subfamily, members of this subfamily do not have inhibitory ankyrin repeats in their C-terminal but are inhibited instead by IκB-like proteins, such as Cactus. All members of this superfamily form homodimers and heterodimers to function and selectively bind to their DNA targets (Gilmore and Wolenski 2012). Aside from NF-κB, members of the NFAT protein family are also RHD-containing transcription factors. Four of its members (NFATC1-4) are regulated by calcium signaling while NFAT5 is the only member that is non-calcium-regulated and is involved in osmotic stress responses. Most members of this protein family are also involved in immune response (Macian 2005). It has been hypothesized that the NFAT family evolved from the NF-κB proteins before the split of Cnidaria and Bilateria (Gilmore and Wolenski 2012).

**Figure 4:**
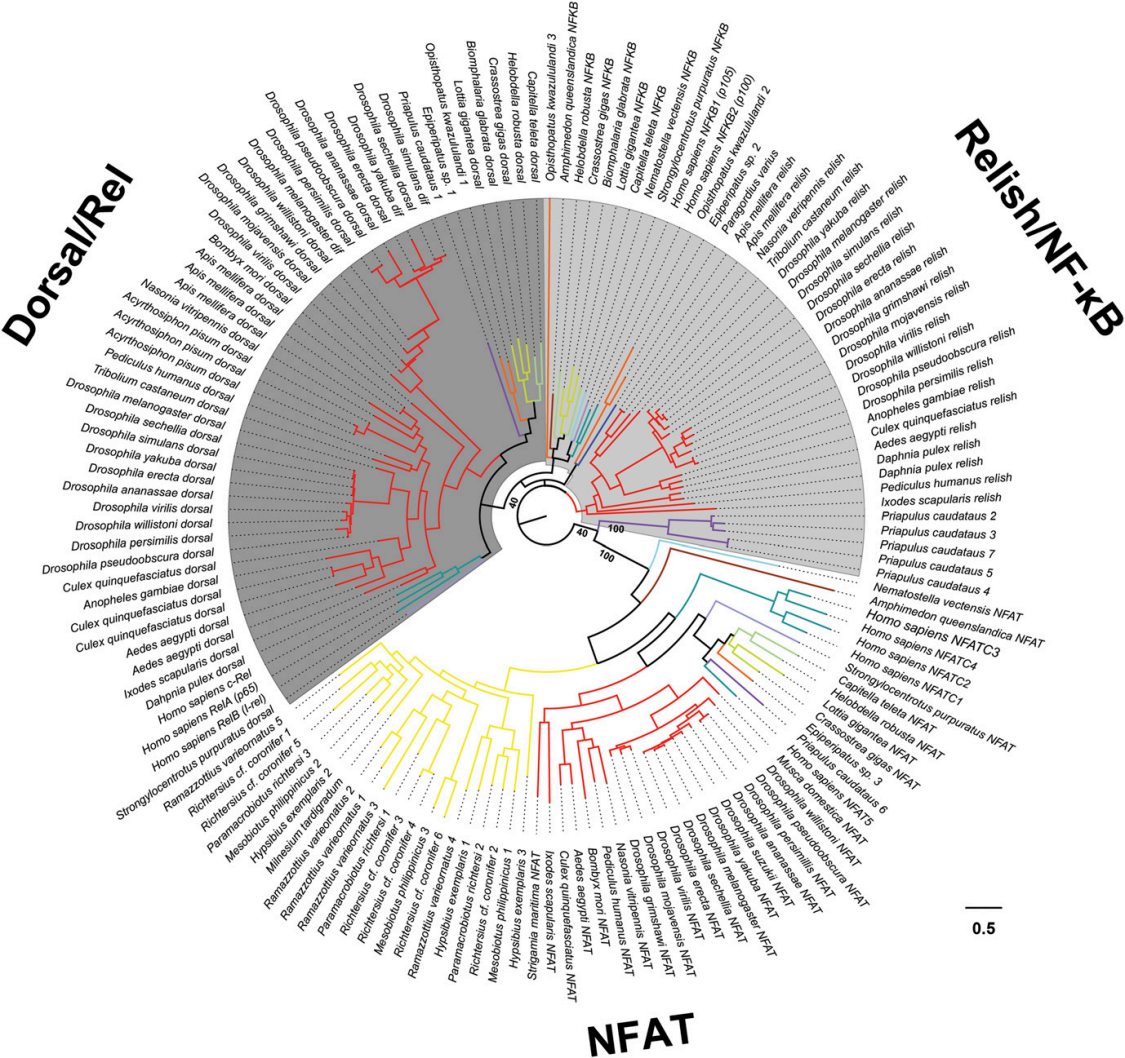
Phylogenetic tree of Rel homology domain (RHD)-containing proteins in the 1^st^ set of candidate genes of tardigrades and non-arthropod ecdysozoans, together with Dif, Dorsal, Relish, and NFAT orthologs of poriferans, cnidarians, arthropods, mollusks, annelids, echinoderms, and chordates. The tree was built using the VT+G+I model with 1000 bootstrap replicates and midpoint rooted. Branch colors represent different phyla. Complete bootstrap values available in Figure S2. Scale bar = number of nucleotide substitution/site.

Out of all the ecdysozoans we analyzed, only the tardigrades lacked detectable *NF-κB* homologs (Figures 1b and 3b, Table S6). However, with our profile HMM search we were still able to identify RHD-containing sequences in all tardigrades except for the heterotardigrades. In order to verify that these sequences do not belong to the NF-κB superfamily, we conducted a phylogenetic analysis using all the RHD-containing sequences from all samples we analyzed, together with members of the NFAT family, NF-κB and Relish subfamilies of the NF-κB superfamily from two early-diverging animal phyla (Porifera and Cnidaria) and multiple Bilateria (Arthropoda, Mollusca, Annelida, Echinodermata and Chordata). The reconstructed tree showed that all the tardigrade RHD-containing domains clustered with the NFAT proteins instead of the NF-κB proteins (Figures 4 and S2). These results further support the absence of *NF-κB* homologs in tardigrades.

If *NF-κB* homologs were truly absent, this would make tardigrades more similar to *C. elegans*, which also lacks *NF-κB* homologs (Irazoqui *et al.* 2010; Brennan and Gilmore 2018). Eutardigrades and *M. tardigradum*, however, still possess putative homologs of the NFAT transcription factors. These proteins might be involved in the tardigrade immune response, as NFAT proteins have immune functional roles in other animal groups (Zanoni and Granucci 2012; Song *et al.* 2013; Huang *et al.* 2015). It must be noted however that the true absence of RHD-containing homologs in heterotardigrades can only be verified once a more complete genome or transcriptome assembly becomes available.

### Tardigrades have a conserved D. melanogaster JNK pathway

**Figure 5:**
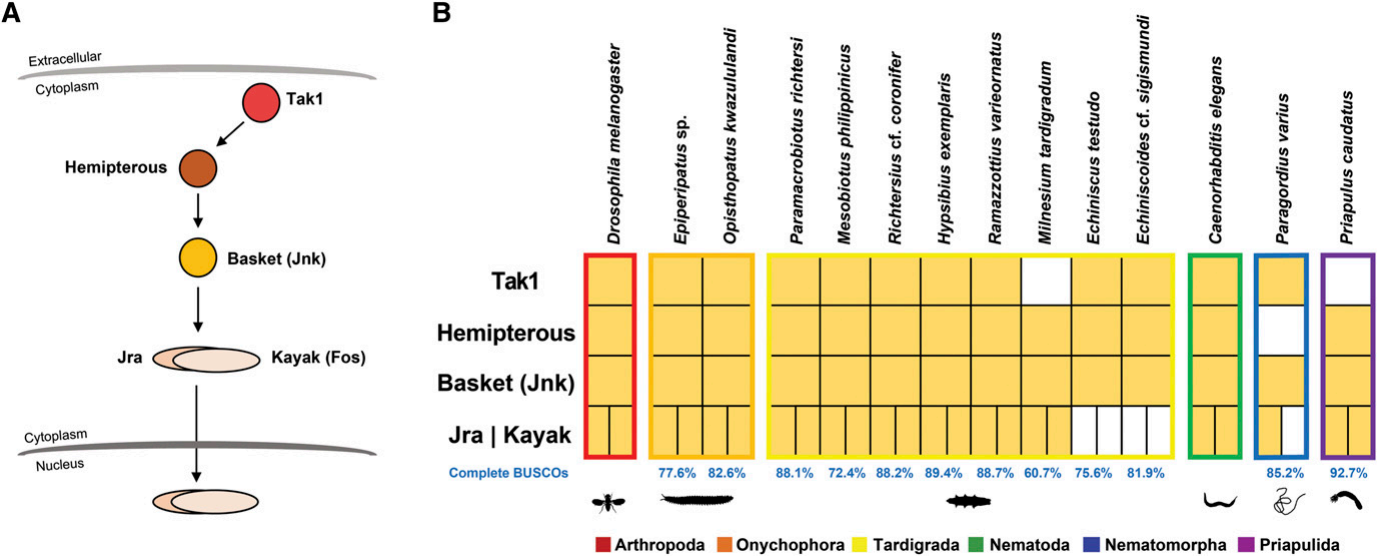
Tardigrade and other ecdysozoan JNK pathway gene homologs. (A) The *Drosophila melanogaster* JNK pathway. (B) The presence (filled boxes) and absence (empty boxes) of *D. melanogaster* JNK pathway gene homologs in tardigrades and other ecdysozoans.

The JNK pathway (Figure 5a) is known to be involved in *D. melanogaster* immune response, specifically in the expression of antimicrobial peptides (Delaney *et al.* 2006). However, this pathway is also involved in other important cellular processes, such as autophagy, apoptosis, metabolism, and growth (Biteau *et al.* 2011). Due to its multiple functions, it is therefore not surprising that this pathway is conserved in most tardigrades (eutardigrades) and other ecdysozoans (Figure 5b). In fact, some functionality of the JNK pathway has been shown to be conserved from Cnidaria to Chordata, since it was shown to prevent oxidative stress caused by UV and thermal stresses in cells of both corals and humans (Courtial *et al.* 2017). It is therefore possible that this pathway is involved in tardigrade UV and thermal stress responses – stresses to which tardigrades are well known to be extremely resilient against (Jönsson *et al.* 2008).

### The Caenorhabditis elegans Tir-1 pathway is conserved in tardigrades

**Figure 6:**
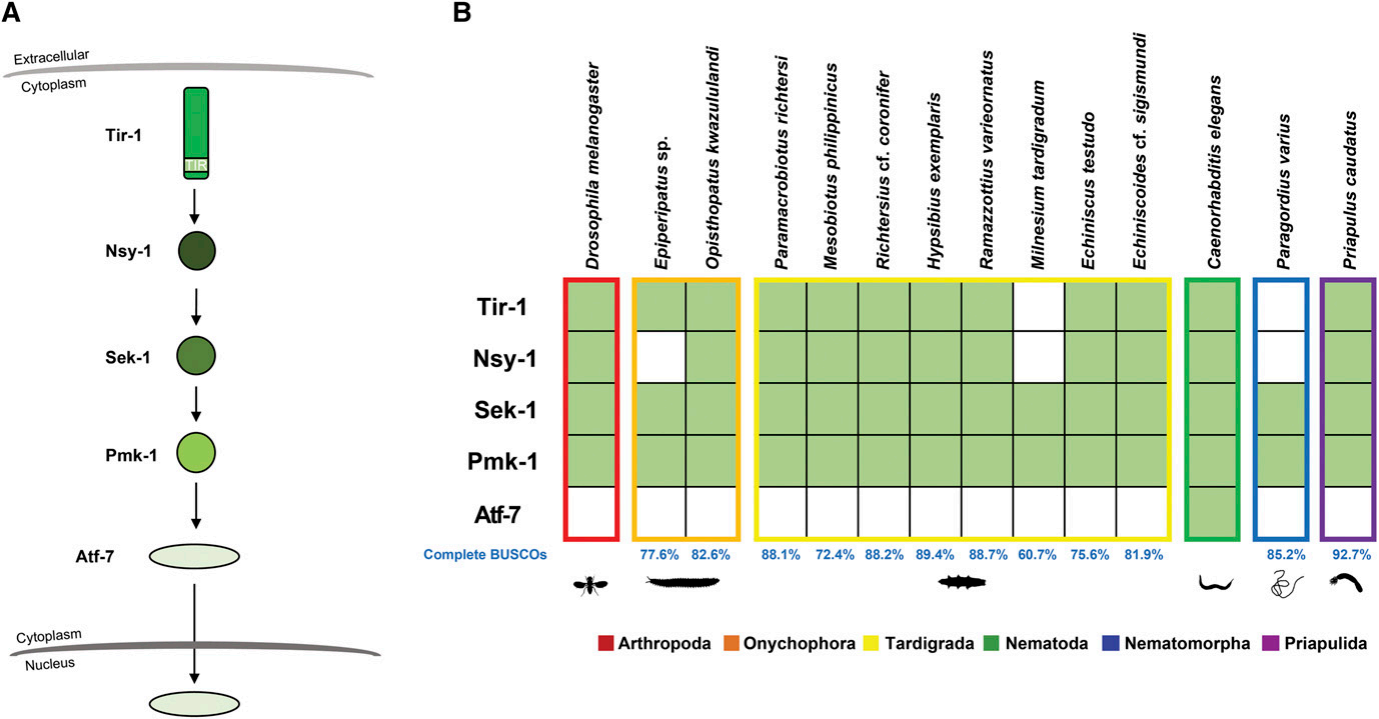
Tardigrade and other ecdysozoan Tir-1 pathway gene homologs. (A) The *Caenorhabditis elegans* Tir-1 pathway. (B) The presence (filled boxes) and absence (empty boxes) of *C. elegans* Tir-1 pathway gene homologs in tardigrades and other ecdysozoans.

In terms of antibacterial immune response, the nematode *C. elegans* is different from *D. melanogaster* and humans since it utilizes a mitogen-activated protein kinase pathway, the Tir-1 pathway (Figure 6a), instead of the canonical Toll pathway and activation of NF-κB transcription factors (Irazoqui *et al.* 2010). Our results showed that putative homologs of the *Tir-1* gene and all the downstream kinases (*Nsy-1*, *Sek-1*, and *Pmk-1*) were present in all tardigrades, except for *M. tardigradum* that had no detectable *Tir-1* and *Nsy-1* homologs (Figure 6b). Homologs of the transcription factor *Atf-7* were not detected in any tardigrade species. For the other ecdysozoans, *Tir-1* and the kinase homologs were also detected in most transcriptomes. *P. varius* showed a similar signature to that of *M. tardigradum*, since no *Tir-1* and *Nsy-1* homologs were detected in these species.

The detection of Tir-1 pathway gene homologs in tardigrades and in other ecdysozoans suggests that these genes are conserved across Ecdysozoa. Since tardigrades do not appear to have homologs of most of the *D. melanogaster* Toll and Imd pathway genes, the Tir-1 pathway may well be an important component of the antimicrobial response in tardigrades. However, the presence of the Tir-1 pathway homologs in tardigrades could also be due to their involvement in other cellular functions. For example, *Ask-1* (the mammalian homolog of *Nsy-1*) has been shown to be involved in multiple stress responses (Hattori *et al.* 2009). Future work will be critical for determining whether the Tir-1 pathway is in fact a necessary component of tardigrade immune response.

### Conclusion

Our results show that there is a substantial difference in the antimicrobial recognition and signaling pathways across Ecdysozoa. The two examined onychophorans (which belong to clades that diverged in the Paleozoic; Giribet *et al.* 2018) generally showed the same pattern present in as *D. melanogaster*, except in the Imd pathway. Tardigrades, on the other hand, seemed to have an immune response gene repertoire more similar to nematoids and priapulans and quite distinct from that of other panarthropods, wherein homologs of most of the *D. melanogaster* immune genes were not detected. Thus, if these genes are truly absent, immune gene loss occurred not only in *C. elegans* but also in other ecdysozoan lineages, suggesting that these events may be more common within the group than previously thought. Furthermore, depending on the accepted phylogeny (see Giribet & Edgecombe 2017), these gene losses could have occurred in the common ancestor of tardigrades and cycloneuralians or independently within each phylum. Since Toll or TLR pathway components are conserved across sponges and humans (Brennan and Gilmore 2018), it is interesting to note that at least four ecdysozoan phyla lack components of this pathway. This raises the question of why these gene losses occurred within these particular ecdysozoans. However, it should be noted that although all transcriptomes showed relatively high complete BUSCO values, this does not necessarily mean that the missing homologs of the immune pathways were absent in the genome, as these genes could have been missed in the dataset due to low-expression (or non-expression) since the samples were not infected. Future experimental work involving differential gene expression analysis during pathogen infection are therefore required to validate the results we have obtained.

The tardigrade species we examined showed similar patterns in terms of the absence and presence of immune-related *D. melanogaster* and *C. elegans* gene homologs. However, it is worth noting that the tardigrades used in this study are all limno-terrestrial in origin, except for *E.* cf. *sigismundi*. The gene repertoire of the immune system of other marine tardigrades needs to be examined in order to determine if these characteristics are general features of the phylum Tardigrada. Nevertheless, our results suggest that tardigrades use a different antimicrobial pathway than other panarthropods, since tardigrades lack components of the NF-κB signaling pathway. These losses could have been caused by the reduction of genome complexity in tardigrades due to their miniaturization (Gross *et al.* 2019) Indeed, the same pattern of gene losses was observed in tardigrade Hox genes (Smith *et al.* 2016). Furthermore, pathways that have multiple functions (*e.g.*, in immunity and stress-response), such as the JNK and Tir-1 pathways, could have been selectively retained. Experimental studies can also be performed to determine if these tyrosine kinase pathways are involved in the tardigrade immune response and if tardigrades possess novel immune mechanisms and antimicrobial peptides. Overall, this study provides a framework for future studies to elucidate how immune systems function in these extremely resilient organisms.
